# Genetic Architecture of Maize Stalk Diameter and Rind Penetrometer Resistance in a Recombinant Inbred Line Population

**DOI:** 10.3390/genes13040579

**Published:** 2022-03-24

**Authors:** Huanhuan Liu, Huan Wang, Cong Shao, Youle Han, Yonghui He, Zhitong Yin

**Affiliations:** 1 Jiangsu Key Laboratory of Crop Genomics and Molecular Breeding/Key Laboratory of Plant Functional Genomics of the Ministry of Education/Jiangsu Key Laboratory of Crop Genetics and Physiology, Agricultural College of Yangzhou University, Yangzhou 225009, China; liuhh@yzu.edu.cn (H.L.); whgenes@163.com (H.W.); shaocongxoxo@126.com (C.S.); bana0221@163.com (Y.H.); heyonghui@yzu.edu.cn (Y.H.); 2 Jiangsu Co-Innovation Center for Modern Production Technology of Grain Crops, Yangzhou University, Yangzhou 225009, China

**Keywords:** maize, stalk lodging, stalk diameter, rind penetrometer resistance, QTL mapping

## Abstract

Stalk lodging presents a major constraint on maize (*Zea mays* L.) quantity and quality and hampers mechanized grain harvesting. Stalk diameter (SD) and rind penetrometer resistance (RPR) are crucial indicators of stalk lodging. To dissect the genetic architecture of these indicators, we constructed a recombinant inbred line (RIL) population derived from a cross between maize inbred lines LDC-1 and YS501 to identify quantitative trait loci (QTLs) controlling SD and RPR. Corresponding phenotypes of basal second, third, and fourth internodes in four environments were determined. By integrating QTL mapping results based on individual environments and best linear unbiased prediction (BLUP) values, we identified 12, 12, and 13 QTLs associated with SD and 17, 14, and 17 associated with RPR. Each QTL accounted for 3.83–21.72% of phenotypic variation. For SD-related QTLs, 30 of 37 were enriched in 12 QTL clusters; similarly, RPR-related QTLs had 38 of 48 enriched in 12 QTL clusters. The stable QTL *qSD9-2* for SD on chromosome 9 was validated and delimited within a physical region of 9.97 Mb. Confidence intervals of RPR-related QTLs contained 169 genes involved in lignin and polysaccharide biosynthesis, with 12 of these less than 500 kb from the peak of the corresponding QTL. Our results deepen our understanding of the genetic mechanism of maize stalk strength and provide a basis for breeding lodging resistance.

## 1. Introduction

As with other high-stalk crops, extreme conditions during the growth period of maize (*Zea mays* L.) can cause lodging, which seriously affects yield and quality. In China, approximately 100 million tons of maize production is lost annually to lodging [1]. This lodging can be subdivided into root and stalk lodging; 30–60% is stalk lodging, which typically leads to more serious yield losses [2,3]. Each year, yield losses from stalk lodging are estimated at 5–20% worldwide [4]. In addition to grain loss, stalk lodging can also disrupt normal plant architecture and damage leaves and stems, thereby aggravating the occurrence of diseases and insect pests and reducing the efficiency of mechanical harvesting [5,6,7]. Therefore, it is imperative to improve stalk lodging resistance in maize.

Establishing an effective and accurate evaluation method for stalk lodging resistance is essential for improving maize stalk strength. Stalk lodging in maize is determined by internal factors, such as plant morphology and stalk strength, as well as external factors such as cultivation management, wind, rain, stalk rot diseases, and insect pests [8,9,10]. The morphological traits that influence the likelihood of stalk lodging include ear height, length and diameter of basal internodes, ear ratio (ear height/plant height), and gravity ratio (center of gravity height/plant height) [11,12,13]. Notably, the contribution of stalk diameter (SD) to lodging resistance is greater than that of basal internode length or ear height [14]. Varieties with thick (i.e., large SD) basal internodes usually have better lodging resistance, with the diameter of the third basal internode being positively correlated with lodging resistance [15,16]. In addition to plant morphology, stalk mechanical traits, such as rind penetrometer resistance (RPR), stalk breaking force (SBF), and stalk bending strength (SBS), are positively related to lodging resistance [6,17,18,19,20]. Rind penetrometer resistance refers to the force required to pierce the stalk rind with a spike attached to a digital force gauge [21]. With the advantage of fast, convenient, and non-destructive measurement, RPR is a common metric for evaluating stalk strength [6,21,22,23,24]. As a consequence, SD and RPR have become critical indicators of stalk lodging resistance.

Stalk diameter and RPR are complex quantitative traits controlled by multiple loci. In recent years, many related quantitative trait loci (QTLs) and quantitative trait nucleotides (QTNs) have been detected using linkage analysis and genome-wide association studies (GWAS) under different environmental conditions. Joint-linkage QTL mapping and GWAS in a panel of 2453 diverse inbreds from the North Central Regional Plant Introduction Station (NCRPIS) identified 18 family-nested QTLs and 141 significant GWAS associations controlling RPR in the NAM and IBM families, and numerous weak associations were also identified [25]. Seven QTLs contributing to RPR variation were detected in two recombinant inbred line (RIL) populations, with the major QTL, *qRPR3-1*, finally delimited into a 3.1-Mb interval [6]. Through multi-locus GWAS for stalk lodging resistance related traits, Zhang et al. identified 29, 34, and 48 QTNs commonly related to SD, SBS, and RPR, respectively, across multiple methods or environments [20]. Their group conducted further genetic analysis on these three traits using a biparental population, identifying 44 QTLs, of which 9 loci contained 13 stalk-lodging–related single-nucleotide polymorphisms (SNPs), as reported in their previous work [10]. Using meta-QTL analysis, 95 QTLs for maize stem diameter from 17 different populations were integrated into the IBM Neighbor2 2008 high-density molecular marker linkage map, and 20 meta-QTLs with high consistency were obtained [26]. Liu et al. detected 66 and 45 QTLs, respectively, by genome-wide QTL scanning of RPR from two RIL populations at seven growth stages [27]. However, few relevant genes for SD and RPR have been cloned, owing to their complex genetic basis and the strong influence of environmental conditions and genetic backgrounds.

Genes controlling other stalk-lodging–related traits, such as cell wall composition, stalk strength, and plant height, have also been identified. The gene *stiff1*, underlying a major QTL for stalk bending strength, has been cloned [28]. Related genes of brittle stalk mutants (*bk2* and *bk4*) and brown midrib mutants (*bm1-5*) are considered to be involved in the regulation of cellulose and lignin biosynthesis of the stalk [29,30,31,32,33,34,35]. Some mutations related to plant height and internode length, such as *an1* [36], *dwarf3* [37], *dwarf8* [38], *bv1* [39], *br2* [40,41], and *brd1* [42], are also associated with stalk lodging resistance. In addition, several transcription factors and small RNAs are involved in regulating stem strength [43,44]. However, most of these genes have been cloned from mutants and are often accompanied by severe negative effects on yield and other agronomic traits, greatly limiting their application in maize breeding. Therefore, it is essential to further analyze the genetic basis of stalk lodging resistance and explore more functional genes.

In this study, we constructed an RIL population using the parents YS501 and LDC-1 of the excellent maize variety Tianyu 88 cultivated by our group. Compared with YS501, LDC-1 has a thicker stem diameter and stronger lodging resistance. To dissect the genetic underpinnings of SD and RPR, we collected phenotypic data of the RIL population and its parents under four conditions. The objectives of this study were to (1) estimate the genetic variance and heritability of SD and RPR for the basal second, third, and fourth internodes, as well as the correlation between traits; (2) detect QTLs for SD and RPR using individual environmental analysis and joint analysis in multiple environments; (3) validate and fine-map stable QTL.

## 2. Materials and Methods

### 2.1. Plant Materials

Two excellent maize inbred lines, YS501 and LDC-1, independently cultivated by our group, were used to construct a biparental population of 186 RILs. Each plant from the RIL population was grown in the following environments: Hainan Ledong Experimental Base (N: 18.73°, E: 109.17°) in the winter of 2019 (E1); the experimental field of the Agricultural College of Yangzhou University (N: 32.40°, E: 119.40°) in the spring of 2020 (E2); an experimental field on the Yangzijin campus of Yangzhou University (N: 32.40°, E: 119.40°) in the summer of 2020 (E3); Hainan Ledong Experimental Base (N: 18.73°, E: 109.17°) in the winter of 2020 (E4). The RILs were planted in a randomized complete-block design with two replications. For each accession, 10 plants were planted in a 60 × 20 cm plot. Field management was carried out according to standard agronomic practices. 

### 2.2. Phenotyping

Stalk diameter and RPR of the parents (YS501 and LDC-1) and their derived RIL population were measured under four environmental conditions (E1–E4). At 30 days after pollination, five plants were randomly selected from each line to measure SD and RPR (the leaf sheath was peeled off) of the basal second, third, and fourth internodes. Stalk diameters were obtained from the middle of the corresponding internode using a digital vernier caliper. Stalk RPR was investigated using a digital force gauge (YYD-1B, Zhejiang Top Cloud-agri Technology Co., Ltd., Hangzhou, China). Stalk diameters of the basal second, third, and fourth internodes were described as SD2, SD3, and SD4, respectively, with the stalk RPR of the corresponding internode described as RPR2, RPR3, and RPR4. To ensure the precision of SD and RPR measurements, each individual plant was measured in triplicate. To reduce environmental influence, the best linear unbiased prediction (BLUP) for all traits was calculated using the lme4 software package in the R package with the following mixed linear model (MLM) [45]: Y_ijk_ = μ + G_i_ + E_j_ + GE_ij_ + R_k_(E) + ε_ijk_; the description of all components agreed with that of Hu et al. [19]. 

### 2.3. Data Analysis and QTL Mapping

Statistical analysis and ANOVA for SD and RPR were performed using SPSS 21.0 software. Pearson correlation analyses between pairs of related traits in the RIL population were performed using R software. The formula for calculating broad-sense heritability (*H*^2^) was as follows: *H*^2^ = Vg/(Vg + Vge/l + Vε/rl), where Vg, Vge, and Vε are the variance components of genotype, genotype by environment interaction, and random error, respectively, and r and l represent the number of replicates and environments, respectively [45]. 

QTL mapping was performed using the composite interval mapping option in WinQTL Cartographer 2.5. The walking speed of the QTL analysis was 1.0 cM. A logarithm of odds (LOD) threshold of 2.5 was used to detect significant QTLs, and 1.5-LOD drop intervals were identified for the prediction of confidence intervals. QTLs were named as follows: q+ the order number of basal internodes (2, 3, or 4) + abbreviation of trait + chromosome number + QTL order based on physical location on the chromosome.

### 2.4. Validation and Fine Mapping of qSD9-2

To validate the results of QTL mapping, Indel markers were designed within or near QTL intervals (see Table S1 for specific information on markers). These Indel markers were used to screen recombinant plants from residual heterozygous lines (RHLs). Self-crossed progeny were planted in the experimental field of Yangzhou University in the spring and summer of 2021 (21C and 21X). At 30 days after pollination, SDs of the third internode of each plant were measured and analyzed. The recombinant-derived progeny test [46] was adopted to fine-map *qSD9-2*. The location of candidate genes was determined by detecting the co-separation of phenotype and heterozygous segment genotype in recombinant progenies. Co-isolation indicated that the candidate gene was located in a heterozygous segment, otherwise, the candidate gene was located in a homozygous segment.

## 3. Results

### 3.1. Phenotypic Variation in SD and RPR

**Figure 1:**
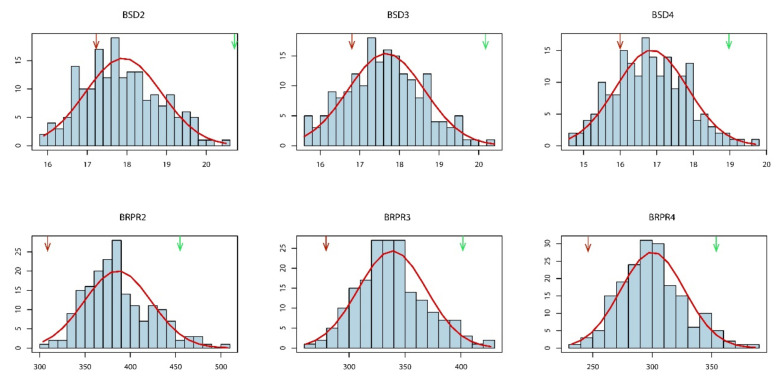
Frequency distributions of BLUP data for SD and RPR in the RIL population. Red and green arrows represent the mean value of the parents YS501 and LDC-1, respectively.

Significant differences in SD and RPR were observed between the two parents under the four environments, with parent LDC-1 having a larger diameter and higher RPR than YS501 (except for SD4 in E2 and RPR4 in E1; Table 1). Descriptive statistical analysis of the RIL population showed that the skewness and kurtosis of all phenotypes were less than 1, indicating that SD and RPR followed a normal distribution (Table 1). The broad-sense heritability of SD2, SD3, and SD4 ranged from 63.77 to 64.38%, while the heritability of RPR was higher, ranging from 78.10 to 83.58%. The phenotypic distribution of the RIL population showed that in some families the mean trait values were greater or less than the mean of either parent, revealing an obvious super-parental phenomenon (Figure 1, Figures S1 and S2). 

**Figure 2:**
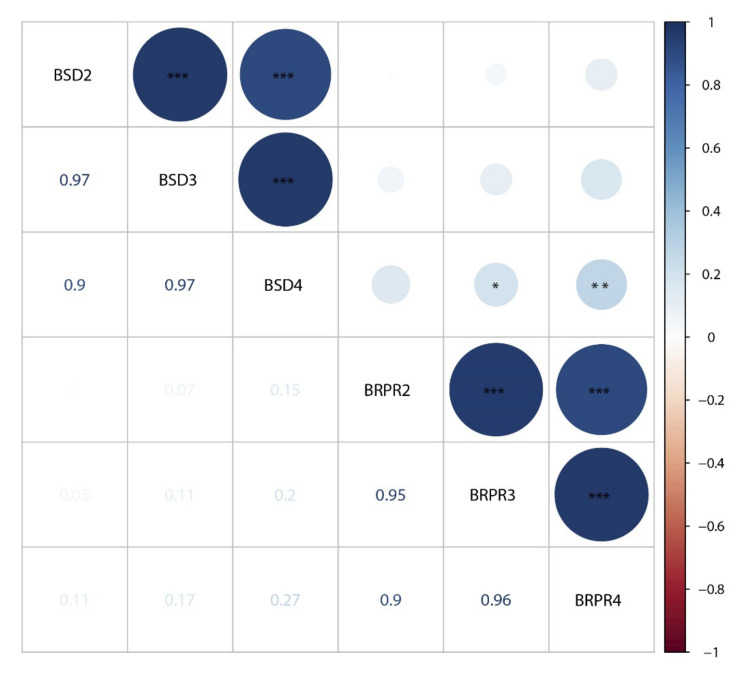
Correlation analysis between SD and RPR based on BLUP values across four environments. Size and color of dots in the upper triangle indicate the degree and direction of correlation, respectively; values in the lower triangle indicate Pearson’s correlation coefficients. BSD2, BSD3, and BSD4 represent BLUP values for SD2, SD3, and SD4, respectively. BRPR2, BRPR3, and BRPR4 represent BLUP values for RPR2, RPR3, and RPR4, respectively. *, **, ***: correlation significant at the 0.05, 0.01, and 0.001 level, respectively.

Correlation analyses were performed among the different traits (Figure 2, Figures S3 and S4). On the basis of the BLUP data, we detected extremely significant positive correlations between the diameters of different internodes (r > 0.9, *p* < 0.001), and the correlation between adjacent internodes (r = 0.97, *p* < 0.001) was even greater. Similarly, correlation coefficients between the RPR of adjacent internodes were 0.95 and 0.96 (Figure 2). However, the correlation between SD and RPR was relatively smaller, and only BSD4 was weakly correlated with BRPR3 (r = 0.2, *p* < 0.05) and BRPR4 (r = 0.27, *p* < 0.01) (Figure 2). For SD (Figure S3) and RPR (Figure S4), it was obvious that correlations between distinct internodes in the same environment were greater than those between the same internodes in distinct environments. In the same environment, the correlation coefficients of SD-related traits were between 0.81 to 0.97, and the correlation coefficients of RPR-related traits were between 0.71 to 0.94. Correlation coefficients of SD2, SD3, SD4, RPR2, RPR3, and RPR4 in different environments were in the range of 0.25–0.41, 0.31–0.43, 0.25–0.47, 0.45–0.58, 0.45–0.54, and 0.43–0.50, respectively. This suggests that the environment had a greater impact on SD and RPR than the location of internodes (at least for these three internodes studied).

### 3.2. QTL Mappings of Stalk Diameters across Four Environments and the BLUP Model

In our previous study, we constructed a high-density linkage map of the RIL population with 2624 bin markers. The map spanned 2569.89 cM, and the average genetic distance between two markers was 0.98 cM (map data are available on the figshare website at the following link: https://figshare.com/articles/dataset/linkage_map_xlsx/19387685, accessed on 21 February 2022).

**Figure 3:**
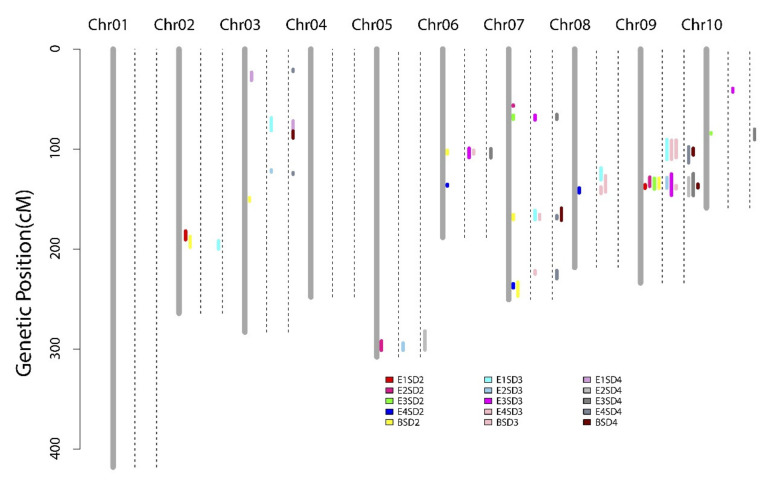
QTL mapping of stalk diameters across four environments and the BLUP model. Colored bars represent QTLs associated with different traits; E1, E2, E3, E4, and B represent the corresponding environment or BLUP model. The two dashed lines next to each chromosome separate the SD-related QTL mapping results for different internodes, SD2, SD3, and SD4, in order. The length of the colored bar indicates the 1.5-LOD QTL support interval.

We identified 12 QTLs for SD2 in the environments and BLUP values, which were distributed on chromosome 2, 3, 5, 6, 7, 8, 9, and 10 (Table 2, Figure 3). Each QTL accounted for 4.19–18.09% of the phenotypic variation, with LOD values ranging from 2.58 to 9.69. The major QTL—*q2SD9-1* on chromosome 9—was repeatedly mapped in four of five datasets (E1, E2, E3, and BLUP) and explained 13.65%, 6.44%, 18.09%, and 9.99% of the phenotypic variation, respectively. Further, *q2SD9-1* exhibited negative additive effects, indicating that the SD-increasing effect of this locus came from parent YS501. Two other QTLs—*q2SD2-1* on chromosome 2 (E1 and BLUP) and *q2SD7-4* on chromosome 7 (E4 and BLUP)—were also discovered in at least two datasets.

The QTL analysis revealed that SD3 was controlled by 12 QTLs (Table 2, Figure 3). Among them, five loci were repeatedly detected in multiple environments or across environments and the BLUP model, while the others could be regarded as environment-specific QTLs. QTL *q3SD6-1*, located on chromosome 6, was co-detected in the E3 and BLUP datasets and explained 5.98% and 5.29% of the phenotypic variation, respectively. QTL *q3SD7-2* was discovered in the E1 and BLUP datasets, with corresponding LOD values of 3.37 and 5.30. QTLs *q3SD8-1* and *q3SD9-1* were identified simultaneously in the E1, E4, and BLUP datasets. The last common QTL, *q3SD9-2*, accounted for 8.18%, 16.84%, and 6.67% of phenotypic variation in the E2, E3, and BLUP datasets, respectively.

In total, 13 QTLs affecting SD4 were detected on chromosomes 2, 3, 5, 6, 7, 9, and 10 across the four environments and the BLUP model, accounting for 4.62–21.72% of the phenotypic variation (Table 2, Figure 3). The major QTL, *q4SD9-2*, which was co-detected across the E2, E3, and BLUP datasets, contributed to 11.55%, 21.72%, and 4.62% of phenotypic variation, respectively. Five (*q4SD2-1*, *q4SD3-1*, *q4SD5-1*, *q4SD9-1*, and *q4SD9-2*) of the QTLs detected for SD4 had negative effects, ranging from −0.26 to −0.82, suggesting that alleles from parent YS501 contributed to increasing the diameter of the basal fourth internode.

Integrating QTL mapping results for SD2, SD3, and SD4 (Figure 3, Table S2), we observed that the QTLs mentioned above were significantly enriched in some chromosomal regions, such as 181.90–199.90 cM on chromosome 2, 99.10–108.70 cM on chromosome 6, 161.00–171.10 cM on chromosome 7, and 124.50–146.40 cM on chromosome 9. Considering QTLs with overlapping confidence intervals as QTL clusters, we identified 12 QTL clusters related to SD, with seven QTLs detected only once in this study. 

### 3.3. QTL Mapping of RPR across Four Environments and the BLUP Model

**Figure 4:**
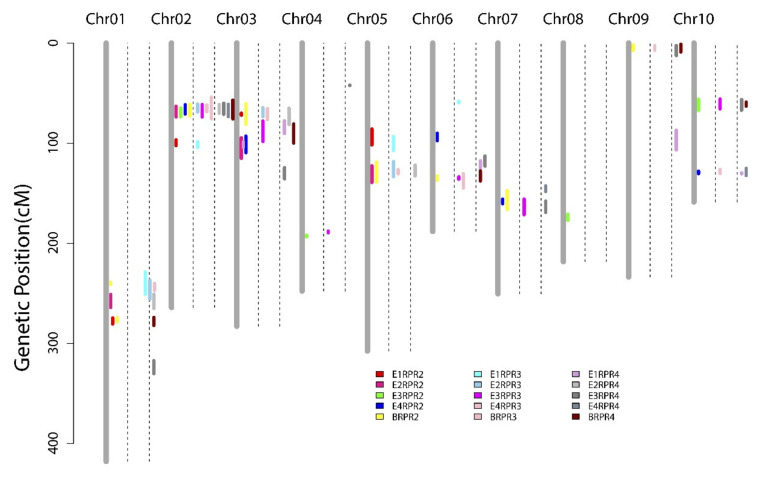
QTL mapping of rind penetrometer resistance across four environments and the BLUP model. Colored bars represent QTLs associated with different traits; E1, E2, E3, E4, and B represent the corresponding environment or BLUP model. The two dashed lines next to each chromosome separate the RPR-related QTL mapping results for different internodes, RPR2, RPR3, and RPR4, in order. The length of the colored bar indicates the 1.5-LOD QTL support interval.

We detected 17 QTLs affecting RPR2 across the four environments and the BLUP model (Table 2, Figure 4). The QTLs detected were distributed across all 10 chromosomes with LOD values ranging from 2.68 to 7.55. Among these QTLs, six loci were repeatedly mapped. QTL *q2RPR2-1* identified in E2, E3, E4, and BLUP datasets was able to explain 7.66%, 4.92%, 8.94%, and 12.38% of the phenotypic variation, respectively. Thirteen QTLs had beneficial alleles derived from LDC-1 (additive effect > 0), while the other four QTLs (*q2RPR5-1*, *q2RPR5-2*, *q2RPR6-1*, and *q2RPR6-2*) possessed alleles derived fromYS501. 

For RPR3, we detected 14 QTLs on chromosomes 1, 2, 3, 4, 5, 6, 7, 9, and 10, covering genetic distances from 1.3 to 22 cM. These QTLs explained 4.11–17.25% of the phenotypic variation, with LOD values ranging from 2.69–10.19. Among them, *q3RPR2-1* on chromosome 2 had the largest effect, accounting for 11.63–17.25% of the phenotypic variation in E2, E3, E4, and BLUP datasets.

A total of 17 QTLs controlling RPR4 were detected across the four environments and the BLUP dataset, with LOD values ranging from 2.58 to 8.57. Only four loci (*q4RPR5-1*, *q4RPR6-1*, *q4RPR6-2*, and *q4RPR9-2*) had negative additive effects, suggesting that alleles from YS501 contributed to enhancing stalk RPR. Six QTLs (*q4RPR2-1*, *q4RPR3-1*, *q4RPR6-1*, *q4RPR9-1*, *q4RPR10-1*, and *q4RPR10-2*) were repeatedly identified in at least two datasets. Notably, *q4RPR2-1* had the greatest contribution, accounting for 14.11% of variation based on BLUP values.

Similar to SD, mapping results for RPR showed that these QTLs were enriched in regions of some chromosomes, such as 228.90–264.00 cM and 274.10–282.10 cM on chromosome 1, 54.00–75.00 cM and 96.80–104.4 cM on chromosome 2, and 118.4–138.9 cM on chromosome 5 (Table S2, Figure 4). After integrating our QTL mapping results for the different internodes, 38 of 48 QTLs controlling RPR were clustered into 12 chromosomal regions (Table S2). In particular, QTLs in two clusters, Cluster-RPR2-1 and Cluster-RPR3-1, were repeatedly identified to be associated with RPR in multiple environments/internodes. 

### 3.4. Validation and Fine Mapping of qSD9-2

**Figure 5:**
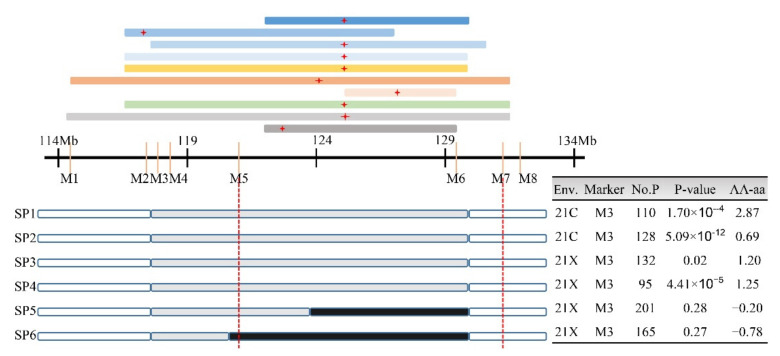
Validation and fine-mapping of *qSD9-2* based on recombinant progeny. QTLs located in this region are represented by colored rectangles above the axis, with red “+” representing the peak of the corresponding QTL. The genetic structure of each recombinant is shown below the axis: SP1-SP6, six recombinants selected from the residual heterozygous line (RHL); white, gray, and black rectangles correspond to homozygous YS501, heterozygous YS501/LDC-1, and homozygous LDC-1 alleles, respectively; Env., environment; Marker, genotype information used for one-way ANOVA; No.P, number of individuals in the secondary population; AA-aa, average SD with YS501 allele–average SD with LDC-1 allele.

SD-related QTLs with the same direction of additive effects were detected 10 times in the region of 114.3–131.5 Mb (Cluster-SD9-2) on chromosome 9, with the peak value of most QTLs at 125.77 Mb (Table S1, Figure 5). Thus, we speculated that they were most likely a common QTL (named *qSD9-2*) that was stable across different environments. To further validate and fine-map the QTL, one RHL that only segregated for the target region was selected using eight indel markers (Table S1). Six recombinants derived from the progeny of these RHLs were used to construct secondary populations in Yangzhou in the spring and summer of 2021 (21C, 21X). The phenotypes of four descendant populations (SP1, SP2, SP3, and SP4) showed co-segregation with the M3 marker, indicating that the candidate gene was located in the heterozygous segment, while the phenotypes of the other two subpopulations (SP5 and SP6) did not co-segregate with genotype, indicating that the candidate gene was located in the homozygous segment (Figure 5). We finally delimited *qSD9-2* to a 9.97-Mb region flanked by markers M5 and M7.

### 3.5. Candidate Gene Prediction of RPR-Related QTLs

Lignin and polysaccharide are considered to be the two key metabolites affecting RPR, and 504 genes related to cellulose and hemicellulose biosynthesis and 341 genes related to lignin biosynthesis have been identified [47]. According to the maize gene annotation database accessible online at MaizeGDB (https://www.maizegdb.org, accessed on 21 February 2022), 169 of these 845 genes were identified in the confidence intervals of RPR-related QTLs (Table S3). Further, we found a total of 12 genes with a distance of less than 500 kb from QTL peaks, which may be candidate genes for these QTLs. The physical interval from *Zm00001d002548* to the *q4RPR2-1* (*q3RPR2-1*/*q2RPR2-1*) peak was 182.8 kb. *Zm00001d002548* encodes an expansin protein, and its homologous gene, *OsEXPA10*, is involved in the coordination of rice development and biotic resistance [48]. *Zm00001d038371*, the ortholog of *LAC17* (*AT5G60020*) in Arabidopsis, was only 96 kb away from the peak of *q4RPR6-1*. *LAC17* encodes a laccase enzyme involved in lignin biosynthesis. Double/triple mutants of *LAC17* and members of its gene family show decreased lignin content in stems and roots [49,50].

## 4. Discussion

### 4.1. Genetic Basis of Lodging-Related Traits at Different Internodes

In the present research, we inferred the genetic architecture of SD and RPR. Broad-sense heritability analysis showed that the heritability of SD (63.77–64.38%) was lower than that of RPR (78.10–83.58%) across the four environments (Table 1). The relatively low heritability of SD has been discovered in several previous studies [20,51], suggesting that SD is more susceptible to environmental changes than RPR. We detected 37 and 48 QTLs associated with SD and RPR, respectively (Table 2). However, only four QTLs (*q2SD9-1*, *q3SD9-2*, and *q4SD9-2* in E3; *q3RPR2-1* based on BLUP values) explained more than 15% of the phenotypic variation, indicating that SD and RPR in maize are regulated by a few major QTLs and multiple loci with minor effects, consistent with the findings of previous studies [4,24,27]. 

Comparing QTL mapping results based on individual environmental and BLUP datasets, we obtained the most QTLs accounting for higher total phenotypic variation using BLUP analysis, consistent with previous genetic detection of maize stalk strength [2,19]. Notably, except for two loci (*q4SD3-3* and *q2SD3-1*), most of the QTLs obtained through joint analysis overlapped with one or more QTLs mapped in an individual environment (Table 2 and Table S2). These results indicate that these loci display strong genetic stability and are not susceptible to environmental effects; they could therefore be used as targets for cloning stalk lodging resistance genes and breeding improved maize in the future. 

Accuracy of phenotypes related to stalk lodging resistance is a prerequisite for QTL mapping and candidate gene mining [7]. We determined the phenotypes of three basal internodes (second, third, and fourth internodes) in four environments in this study. The maize stalk is composed of multiple nodes and internodes, and development of different internodes is not completely synchronized; thus, we speculate that the genetic basis of SD and RPR for different internodes is not completely consistent. The correlation coefficients for both SD and RPR phenotypes between distinct internodes were greater than 0.9, but the correlation coefficients between adjacent internodes were higher (Figure 2, Figures S3 and S4). The most plausible explanation here is the higher synchronicity of development between adjacent internodes. QTL mapping indicated that QTLs for SD and RPR of different internodes overlapped in multiple chromosomal regions (Figure 3 and Figure 4), suggesting that candidate genes in these loci might be continuously effective during maize stalk development. Nevertheless, several QTLs, such as *q4SD2-1*, *q4SD3-1*, *q2SD6-2*, *q4RPR1-1*, and *q4RPR1-3*, were detected only during development of specific internodes. Compared with internode-specific QTLs, the internode-common loci were steadily expressed across environments and could serve as important targets for genetic improvement of maize stalk strength in the future. Therefore, it was reasonable for most researchers to select the SD or RPR of the third internode [23,27,52] or the internode below the primary ear [4,6,23,24,25] to represent the whole plant phenotype in previous studies. However, to achieve a more comprehensive genetic basis for SD and RPR, genetic analysis of the corresponding phenotypes of multiple internodes should be performed.

### 4.2. Pleiotropic QTL Influencing Stalk Lodging Resistance-Related Traits in Maize

Correlation analysis results revealed a low correlation between SD and RPR; only BSD4 showed a significant correlation with BRPR3 (r = 0.2, *p* < 0.05) and BRPR4 (r = 0.27, *p* < 0.01)(Figure 2). Previous studies also showed that SD is only correlated with RPR in individual environments [10,20]. This might be because approximately 50 to 80% of maize strength comes from the rind [53], which has a thickness representing only a fraction of the stalk diameter. However, we still detected overlapping loci controlling the two traits at 25.3–45.7 Mb on chromosome 3 and 155.0–162.2 Mb on chromosome 7 (Figure S5), which might result from tight linkage of candidate genes or pleiotropic effects of a single gene.

### 4.3. Comparison with Previous QTL Mapping Results

In the present study, we detected co-localized QTLs both across environments and across different internodes. This confirms the reliability of our results. To identify the roles of the QTLs identified in other populations, we compared our results with previous QTL mapping and association analyses. Using an RIL population derived from a cross between the B73 and By804 lines, Li et al. detected *qRPR6-1* at 88.5–91.9 cM on chromosome 6 [6], overlapping with our *q4RPR6-1*. Multiple QTLs located on chromosomes 1, 3, 5, 7, and 8 were detected in both our population and an IBM Syn10 DH population analyzed by Zhang et al. [10]. We also discovered two QTLs, *q3RPR4-1* and *q4RPR1-3*, each harboring a significant SNP associated with RPR detected by Zhang et al. [20]. Mazaheri et al. [54] identified 16 candidate genes through genome-wide association analysis of stem-related traits (plant height, diameter, rind thickness, vascular bundle density, and area) in 800 inbred lines. Three of these genes were located within our QTL confidence intervals, namely *Zm00001d031485* (*q3RPR1-1*), *Zm00001d022460* (*q3SD7-3*, *q4SD7-3*), and *Zm00001d044802* (*q2RPR9-1* and *q4RPR9-1*).

### 4.4. Cloned Genes Related to Stalk Phenotype within the QTL Intervals

Several genes affecting cell wall composition and stem morphology and strength have been cloned, most involved in cellulose biosynthesis, plant hormones biosynthesis and transport, and the phenylpropane pathway [7]. Among these cloned genes, six are located within the confidence intervals of our QTLs, namely *Br2*, *Zm4CL1*, *ZmDET2*, *Bm1*, *stiff1*, and *Bk2*. We identified two genes within the confidence intervals of co-localized QTLs *q2RPR1-2* and *q4RPR1-1*. *Br2* participates in polar auxin transport and can significantly reduce internode length below the ear [40]; *Zm4CL1* encodes 4-coumaric acid-CoA ligase 1, and its mutant (*bm5*) shows a decrease in G-type lignin biosynthesis and an increase in soluble ferulic acid derivatives [35]. In the *q2SD3-1* region, *ZmDET2* is involved in the biosynthesis of brassinosterol, and its mutant (*nana plant 1*, *na1*) shows decreased height and extremely shortened internodes [55]. In the Cluster-RPR5-2 region, *Bm1* (*Zm00001d015618*) encodes cinnamyl alcohol dehydrogenase, which shows severely reduced activity after mutation (*bm1*), resulting in changes in both the total amount of lignin and the structure of lignin monomers [31]. We discovered *stiff1*, encoding an F-box domain, in the region of *q3RPR6-1*; inhibition of *stiff1* expression increases the content of cellulose and lignin in the cell wall, thickens stem cells, and enhances stem strength [28]. The stable QTL *qSD9-2* was narrowed down to a 9.97-Mb region that includes *Brittle stalk2* (*Bk2*), which encodes a COBRA-like protein and might play an important role in lignin-cellulose interactions [29]. These genes are likely to be candidate genes for the corresponding QTLs and deserve further attention.

## 5. Conclusions

Stalk lodging seriously affects maize plant type and yield. SD and RPR are closely related to stalk lodging resistance. Properly increasing SD and RPR can significantly increase the mechanical strength of stalks and reduce lodging, thereby achieving high and stable yields. Analyzing the genetic basis of SD and RPR and mining related genes will provide important genetic resources and theoretical support for breeding of maize lodging resistance. In this study, we identified 37 and 48 QTLs related to SD and RPR, respectively, and most of these were grouped in several QTL clusters. The stable QTL *qSD9-2* was fine-mapped and narrowed down to a 9.97-Mb genomic segment. We identified 169 genes associated with cellulose, hemicellulose, and lignin biosynthesis within the confidence intervals of RPR-related QTLs, and 12 of these were less than 500 kb away from the QTL peak. In the future, further fine mapping of these stable QTLs and functional validation of candidate genes will become the focus of our research. At the same time, these stable QTLs could be used for the cultivation of lodging-resistant maize. 

## Figures and Tables

**Table 1:** Phenotypic variation in the recombinant inbred line (RIL) population and its parents.

Trait	Env.	Parent		RIL Population					
		YS501	LDC-1	Mean ± SD^a^	Skew	Kurtosis	Range	CV (%)	H^2^ (%)
SD2	E1	18.74 ± 1.35	21.08 ± 1.63 **	18.94 ± 2.40	0.06	−0.32	13.10–25.50	12.67	63.77
	E2	17.99 ± 2.30	20.17 ± 1.16 *	18.91 ± 3.09	0.33	0.50	10.20–28.60	16.35	
	E3	14.73 ± 0.49	19.68 ± 1.42 **	14.68 ± 1.73	0.13	−0.03	9.60–19.40	11.79	
	E4	18.26 ± 1.48	25.10 ± 0.48 **	18.98 ± 2.67	0.10	−0.02	10.80–25.90	14.06	
	BLUP	17.25 ± 2.25	20.93 ± 2.15 **	17.89 ± 2.88	0.21	−0.30	11.50–27.80	16.07	
SD3	E1	18.14 ± 1.96	19.82 ± 1.31 *	17.97 ± 2.45	−0.18	−0.13	10.70–24.20	13.62	63.82
	E2	17.91 ± 1.82	19.68 ± 0.88 **	18.72 ± 3.01	0.43	0.72	11.00–29.40	16.07	
	E3	14.17 ± 0.57	19.30 ± 0.78 **	14.91 ± 1.71	0.17	0.17	9.70–21.00	11.46	
	E4	18.16 ± 1.28	24.58 ± 0.47 **	18.98 ± 2.69	0.13	−0.02	11.80–26.80	14.17	
	BLUP	16.86 ± 2.34	20.23 ± 1.94 **	17.66 ± 2.73	0.30	−0.14	11.10–26.80	15.49	
SD4	E1	15.44 ± 2.00	16.66 ± 1.21 *	16.11 ± 2.70	−0.15	−0.03	7.50–23.70	16.74	64.38
	E2	17.59 ± 2.21	19.02 ± 0.80	18.19 ± 3.04	0.51	1.17	9.80–30.60	16.69	
	E3	14.53 ± 0.27	19.82 ± 1.31 **	14.87 ± 1.64	0.05	−0.28	10.00–19.10	11.05	
	E4	17.74 ± 1.31	23.46+ ± 0.54 **	18.18 ± 2.53	0.22	−0.04	11.50–25.40	13.94	
	BLUP	16 ± 2.09	19.11 ± 2.38 **	16.85 ± 2.63	0.35	0.19	7.50–25.50	15.59	
RPR2	E1	318.65 ± 40.5	382.86 ± 48.44 **	328.67 ± 53.30	0.18	−0.21	189.10–456.80	16.22	83.58
	E2	318.92 ± 31.84	446.87 ± 48.25 **	392.21 ± 67.87	0.29	0.45	163.00–608.50	17.30	
	E3	304.9 ± 19.96	556.19 ± 46.62 **	448.23 ± 73.33	0.80	0.53	286.20–684.20	16.36	
	E4	271.8 ± 14.75	413.36 ± 26.92 **	376.89 ± 54.43	0.71	0.87	253.60–586.00	14.44	
	BLUP	308.48 ± 33.10	456.52 ± 84.00 **	386.32 ± 69.19	0.47	0.48	190.60–659.70	17.91	
RPR3	E1	294.03 ± 30.18	322.2 ± 47.81 *	295.71 ± 50.15	0.28	0.34	166.40–457.00	16.96	79.58
	E2	285.45 ± 45.04	395.10 ± 33.05 **	342.35 ± 65.89	0.38	0.36	152.80–546.50	19.25	
	E3	278.88 ± 20.15	512.06 ± 34.15 **	397.78 ± 60.30	0.58	0.23	254.30–605.80	15.16	
	E4	249.48 ± 15.37	372.32 ± 26.40 **	319.12 ± 45.51	0.49	0.33	216.10–481.10	14.26	
	BLUP	281.45 ± 32.82	405.74 ± 85.33 **	306.87 ± 53.85	0.61	0.77	179.10–488.70	17.55	
RPR4	E1	259.65 ± 42.31	245.94 ± 41.18	271.05 ± 46.40	0.29	0.31	129.70–420.50	17.12	78.10
	E2	225.74 ± 31.21	356.59 ± 22.67 **	302.01 ± 60.10	0.71	0.65	167.60–519.70	19.90	
	E3	252.16 ± 14.50	464.58 ± 33.61 **	351.32 ± 53.40	0.51	0.60	225.30–548.40	15.20	
	E4	232.22 ± 12.87	330.78 ± 18.68 **	277.64 ± 38.22	0.40	0.13	180.10–401.30	13.77	
	BLUP	245.13 ± 32.10	357.59 ± 86.80 **	300.71 ± 54.43	0.65	0.56	157.50–519.10	18.10	

SD^a^, standard deviation; CV, coefficient of variation; H^2^, broad-sense heritability; SD2, SD3, and SD4, represent the stalk diameter of the basal second, third, and fourth internodes, respectively; RPR2, RPR3, and RPR4, represent rind penetrometer resistance of the basal second, third, and fourth internodes, respectively; Env., specific environment; E1, E2, E3, and E4 represent Hainan in 2019, Yangzhou in the spring of 2020, Yangzhou in the summer of 2020, and Hainan in 2020, respectively; BLUP, best linear unbiased prediction; * and **, indicate significant differences between YS501 and LDC-1 at the 0.05 and 0.01 probability levels, respectively.

**Table 2:** Quantitative trait loci (QTLs) detected for SD and RPR across four environments and the BLUP model.

Trait	QTL	Chr	Env.	LOD	PVE (%)	A	Peak (cM)	Interval (CM)	Peak (Mb)	Physical Location (Mb)
SD2	*q2SD2-1*	2	E1	3.42	6.86	−0.60	182.61	181.90–190.60	215.37	215.16–220.88
			BLUP	2.82	4.89	−0.22	194.51	187.40–198.10	224.43	217.21–225.33
	*q2SD3-1*	3	BLUP	3.07	4.82	0.21	150.31	148.50–152.00	182.51	181.14–183.88
	*q2SD5-1*	5	E2	3.49	7.16	−0.65	294.31	291.80–301.20	220.94	220.49–221.52
	*q2SD6-1*	6	BLUP	3.24	5.14	0.22	101.91	101.10–104.90	144.58	143.82–151.51
	*q2SD6-2*	6	E4	2.68	5.06	0.53	135.81	135.20–136.90	162.43	161.55–162.81
	*q2SD7-1*	7	E2	2.58	5.17	−0.55	56.51	56.00–57.00	8.76	8.52–9.14
	*q2SD7-2*	7	E3	3.13	5.38	0.35	68.81	66.20–70.10	15.41	13.64–18.33
	*q2SD7-3*	7	BLUP	4.86	7.93	0.27	167.21	165.50–170.50	161.42	159.78–162.19
	*q2SD7-4*	7	E4	3.28	6.25	0.61	236.51	234.50–238.80	177.96	177.67–178.65
			BLUP	3.64	5.93	0.24	236.51	233.00–246.90	177.96	178.65–180.75
	*q2SD8-1*	8	E4	2.84	4.19	0.20	141.81	138.70–143.80	162.92	161.26–164.25
	*q2SD9-1*	9	E1	6.51	13.65	−1.16	136.31	135.60–139.10	125.77	121.73–129.87
			E2	3.16	6.44	−0.61	129.41	128.00–137.20	117.68	117.07–127.65
			E3	9.69	18.09	−0.63	136.31	129.20–139.90	125.77	117.68–130.34
			BLUP	6.03	9.99	−0.31	136.31	129.00–139.00	125.77	117.07–129.87
	*q2SD10-1*	10	E3	2.83	4.85	0.34	83.61	83.60–84.70	133.44	133.44–138.54
SD3	*q3SD2-1*	2	E1	3.13	6.16	−0.59	195.51	191.60–199.90	224.43	221.4–226.88
	*q3SD3-1*	3	E1	2.92	5.46	0.56	72.01	68.80–81.40	30.98	25.35–45.72
	*q3SD3-2*	3	E2	4.48	9.26	0.77	122.01	120.80–122.80	167.66	164.41–168.35
	*q3SD5-1*	5	E2	3.15	6.32	−0.60	299.41	293.80–301.20	221.36	220.49–221.52
	*q3SD6-1*	6	E3	3.70	5.98	0.36	103.81	99.10–108.40	149.26	141.26–152.61
			BLUP	3.34	5.29	0.22	103.81	101.10–104.90	149.26	143.82–151.51
	*q3SD7-1*	7	E3	3.35	5.39	0.34	68.81	66.00–70.80	15.41	13.64–21.49
	*q3SD7-2*	7	E1	3.37	6.31	0.59	167.21	161.30–170.50	161.42	158.52–162.19
			BLUP	5.30	8.66	0.29	167.21	165.40–170.30	161.42	159.78–162.19
	*q3SD7-3*	7	E4	3.39	6.34	0.61	222.21	221.50–225.00	175.02	175.63–176.82
	*q3SD8-1*	8	E1	3.36	6.34	0.61	119.31	118.80–130.60	145.98	145.83–154.57
			E4	2.92	5.46	0.55	141.71	137.90–144.40	162.92	161.02–164.25
			BLUP	3.29	6.17	0.59	128.71	126.60–142.70	150.93	150.21–163.87
	*q3SD9-1*	9	E1	4.53	8.42	−0.68	100.81	90.40–110.40	85.54	36.17–103.21
			E4	3.78	7.14	−0.65	101.41	91.20–110.20	92.35	38.64–103.21
			BLUP	5.09	8.14	−0.35	100.81	91.00–108.60	85.54	37.79–100.82
	*q3SD9-2*	9	E2	4.06	8.18	−0.68	136.31	128.40–139.00	125.77	117.07–129.87
			E3	9.70	16.84	−0.60	135.81	124.80–146.20	124.08	114.3–131.5
			BLUP	3.97	6.67	−0.42	137.11	136.40–139.70	127	125.77–129.87
	*q3SD10-1*	10	E3	3.12	5.01	0.33	40.31	39.30–42.90	8.79	5.9–10.42
SD4	*q4SD2-1*	2	E1	5.10	9.59	−0.77	25.71	23.10–31.20	4.28	3.88–6.16
	*q4SD3-1*	3	E4	2.82	4.76	−0.51	21.31	20.30–22.30	3.14	3.09–4.76
	*q4SD3-2*	3	E1	2.76	5.47	0.56	72.01	71.80–80.50	30.98	29.43–44.15
	*q4SD3-3*	3	BLUP	3.29	5.68	0.24	85.91	81.90–88.90	55.4	45.73–102.71
	*q4SD3-4*	3	E4	2.70	4.94	0.50	124.21	123.50–125.10	168.35	167.66–168.77
	*q4SD5-1*	5	E2	3.05	7.13	−0.60	294.31	281.80–300.90	220.94	218.52–221.52
	*q4SD6-1*	6	E3	3.63	5.41	0.33	103.81	99.60–108.70	149.26	141.26–152.61
	*q4SD7-1*	7	E3	3.59	5.33	0.33	68.81	65.30–70.20	15.41	13.64–21.49
	*q4SD7-2*	7	E4	3.24	5.92	0.55	167.21	166.50–169.60	161.42	160.9–162.19
			BLUP	5.86	9.70	0.31	167.21	159.10–171.10	161.42	157.09–162.19
	*q4SD7-3*	7	E4	3.25	5.88	0.54	222.21	221.30–229.60	175.02	175.63–179.03
	*q4SD9-1*	9	E4	5.62	10.44	−0.73	101.41	97.60–113.60	91.64	70.77–105.86
			BLUP	3.36	5.32	−0.27	100.81	99.20–105.80	85	82.57–94.9
	*q4SD9-2*	9	E2	5.60	11.55	−0.82	136.31	128.60–146.60	125.77	117.07–131.5
			E3	12.98	21.72	−0.66	136.31	124.50–146.40	125.77	114.3–131.5
			BLUP	2.91	4.62	−0.26	135.81	135.00–138.80	122.42	121.73–129
	*q4SD10-1*	10	E3	4.45	6.69	0.37	83.61	80.00–90.50	133.44	126.32–138.54
RPR2	*q2RPR1-1*	1	BLUP	2.78	4.03	7.52	240.71	238.90–241.30	197.5	194.7–197.84
	*q2RPR1-2*	1	E2	5.40	9.80	17.25	259.41	251.20–264.00	211.12	202.08–214.25
	*q2RPR1-3*	1	E1	5.05	9.04	14.85	275.21	274.60–280.60	220.78	219.09–225.03
			BLUP	2.99	4.31	7.70	275.81	274.40–278.50	222.27	219.09–223.6
	*q2RPR2-1*	2	E2	3.83	7.66	15.58	69.41	63.10–73.70	16.39	12.57–19.34
			E3	2.76	4.92	17.55	72.51	64.90–73.70	18.76	12.57–19.34
			E4	4.88	8.94	14.17	65.01	61.70–70.90	15.01	12.57–18.31
			BLUP	7.55	12.38	13.24	65.01	61.40–72.90	15.01	12.57–19.34
	*q2RPR2-2*	2	E1	3.08	5.42	11.55	99.11	96.80–102.50	43.98	41.24–55.85
	*q2RPR3-1*	3	E1	2.92	5.17	11.61	71.11	70.10–72.00	29.43	27.55–33.2
			BLUP	7.15	11.71	13.19	71.11	60.60–80.90	29.43	16.96–45.43
	*q2RPR3-2*	3	E2	6.51	11.72	20.04	105.91	94.60–115.10	153.57	128.63–163.19
			E4	3.78	6.91	12.70	103.11	93.10–109.40	149.05	119.21–158.12
	*q2RPR4-1*	4	E3	2.76	5.07	17.37	192.61	192.00–193.60	231.9	231.9–235.51
	*q2RPR5-1*	5	E1	4.29	7.65	−13.71	89.81	85.90–101.70	26.06	19.66–36.42
	*q2RPR5-2*	5	E2	6.37	11.37	−18.58	128.91	122.70–139.20	82.97	76.22–145.91
			BLUP	5.27	8.40	−10.97	128.91	119.00–138.90	82.97	68.75–145.91
	*q2RPR6-1*	6	E4	2.76	4.93	−10.49	96.31	90.20–97.50	136.98	133.53–141.26
	*q2RPR6-2*	6	BLUP	3.47	5.41	−8.76	134.71	132.70–137.00	161.55	160.9–162.81
	*q2RPR7-1*	7	E4	3.16	5.66	11.16	159.21	156.00–160.40	157.7	155–158.52
			BLUP	4.76	7.61	10.26	158.51	147.50–166.30	157.7	151.21–160.9
	*q2RPR8-1*	8	E3	3.24	6.39	19.68	174.31	171.10–177.20	173.03	171.99–174.33
	*q2RPR9-1*	9	BLUP	2.89	4.17	7.64	5.31	1.80–7.20	4.44	1.84–5.08
	*q2RPR10-1*	10	E3	3.68	6.71	20.69	60.61	56.20–67.30	39.07	19.92–90.13
	*q2RPR10-2*	10	E4	2.68	4.83	10.47	129.81	128.00–130.50	145.97	145.56–146.56
RPR3	*q3RPR1-1*	1	E1	4.90	8.93	13.25	239.11	228.90–250.90	195.09	186.38–203.7
			E2	4.18	8.05	15.39	253.51	237.00–256.00	204.96	193.34–208.08
			BLUP	2.74	4.24	6.41	240.41	240.10–247.00	197.05	195.68–201.74
	*q3RPR2-1*	2	E2	6.27	12.95	22.55	65.01	61.00–68.90	15.01	15.01–18.31
			E3	7.25	12.47	17.81	65.01	61.50–73.70	15.01	12.57–19.34
			E4	6.34	11.63	12.85	65.01	61.90–68.60	15.01	12.57–18.31
			BLUP	10.19	17.25	12.91	65.01	54.00–75.70	15.01	11.17–19.71
	*q3RPR2-2*	2	E1	3.07	5.58	10.41	99.71	98.70–104.40	49.26	43.54–59.84
	*q3RPR3-1*	3	E2	4.04	7.75	16.32	65.51	64.60–73.50	22.67	21.00–32.39
			BLUP	4.60	7.27	8.58	71.11	65.60–76.50	29.43	27.55–37.81
	*q3RPR3-2*	3	E3	4.36	7.29	13.85	88.41	78.00–98.30	63.86	38.78–140.85
	*q3RPR4-1*	4	E3	2.69	4.41	10.66	188.61	187.90–189.40	230.7	226.19–231.58
	*q3RPR5-1*	5	E1	5.30	9.80	−13.83	104.51	93.40–107.40	41.98	31.81–51.91
	*q3RPR5-2*	5	E2	3.73	7.03	−14.33	128.41	118.40–133.50	82.99	68.35–93.1
			BLUP	2.84	4.11	−6.29	128.91	126.50–130.60	82.97	70.34–88.29
	*q3RPR6-1*	6	E1	2.94	5.31	−10.28	58.81	58.30–59.60	95.99	95.86–98.07
	*q3RPR6-2*	6	E3	2.95	4.85	−11.15	134.71	133.70–136.00	161.55	160.9–162.81
			BLUP	5.64	8.94	−9.27	135.81	130.60–144.80	162.43	159.79–165.36
	*q3RPR7-1*	7	E3	4.38	7.33	13.50	159.21	156.00–171.40	157.7	155.00–162.19
	*q3RPR9-1*	9	BLUP	2.80	4.34	6.45	5.31	2.70–7.20	4.44	3.99–5.08
	*q3RPR10-1*	10	E3	2.94	4.50	10.89	60.61	56.00–65.70	39.07	17.92–86.46
	*q3RPR10-2*	10	E4	2.73	5.07	8.56	127.31	126.20–130.10	145.56	145.47–146.56
RPR4	*q4RPR1-1*	1	E2	3.51	6.38	12.49	260.21	250.90–265.10	210.46	203.7–214.51
	*q4RPR1-2*	1	BLUP	3.60	5.07	6.14	275.81	274.10–282.10	219.76	219.09–227.88
	*q4RPR1-3*	1	E3	3.37	5.38	10.36	327.21	317.40–330.20	262.09	255.17–263.52
	*q4RPR2-1*	2	E2	4.57	8.57	14.76	65.11	61.50–70.00	15.01	12.57–18.31
			E3	5.38	8.92	13.50	65.01	60.10–71.20	15.01	12.57–18.31
			E4	6.29	11.88	11.59	65.01	61.20–73.50	15.01	12.57–19.34
			BLUP	8.57	14.11	10.34	65.01	57.00–75.70	15.01	12.24–19.71
	*q4RPR2-2*	2	E1	4.86	9.37	12.84	99.71	98.70–104.30	49.26	43.54–57.89
	*q4RPR3-1*	3	E1	3.95	7.54	11.86	88.41	77.70–90.30	63.86	38.78–114.25
			E2	4.74	8.73	15.55	71.11	65.10–81.40	29.43	21.00–54.00
			BLUP	5.50	8.66	8.24	89.51	80.90–99.80	108.5	44.15–142.88
	*q4RPR3-2*	3	E3	4.28	6.94	11.70	130.41	124.50–135.60	172.48	168.35–175.09
	*q4RPR4-1*	4	E3	2.58	3.83	8.77	42.21	42.10–42.50	10.45	6.09–10.76
	*q4RPR5-1*	5	E2	5.26	9.51	−15.32	128.91	121.90–132.70	82.97	72.79–95.1
	*q4RPR6-1*	6	E1	3.29	6.11	−10.30	123.41	117.60–125.30	158.32	155.77–159.37
			E3	3.27	5.13	−10.30	116.41	112.80–122.90	155.77	153.76–158.32
	*q4RPR6-2*	6	BLUP	5.08	7.94	−7.69	135.81	127.80–137.80	162.43	159.53–162.81
	*q4RPR7-1*	7	E4	3.38	6.09	8.35	146.81	142.60–148.20	151.21	148.97–152.57
	*q4RPR7-2*	7	E3	3.20	4.71	9.63	159.21	158.00–169.20	157.7	157.09–162.19
	*q4RPR9-1*	9	E3	3.09	4.94	9.82	5.31	2.50–12.50	4.44	1.84–6.79
			BLUP	3.79	5.91	6.55	2.41	1.20–8.80	3.99	2.79–6.69
	*q4RPR9-2*	9	E1	3.77	7.05	−11.06	96.91	87.10–106.80	70.77	30.77–96.81
	*q4RPR10-1*	10	E3	5.57	9.18	13.77	60.61	56.40–67.10	39.07	19.92–90.13
			BLUP	2.84	4.34	5.72	62.81	59.00–63.10	72.23	27.25–76.55
	*q4RPR10-2*	10	E1	2.62	4.58	8.96	129.81	129.70–130.60	145.97	145.97–146.56
			E4	3.70	7.10	9.15	128.31	125.30–132.30	145.97	145.22–146.56

Chr, chromosome. LOD, logarithm of odds. PVE, Phenotypic variation explained by each QTL. A, Additive effect. Interval, confidence interval.

## Data Availability

The genetic map data are available on the figshare website at the following link: https://figshare.com/articles/dataset/linkage_map_xlsx/19387685, accessed on 21 February 2022.

## Supplementary Materials

The following are available online at https://www.mdpi.com/article/10.3390/genes13040579/s1, Figure S1: Frequency distributions of SD in the RIL population in four environments; Figure S2: Frequency distributions of RPR in the RIL population in four environments; Figure S3: Correlation analysis of SD in all environments; Figure S4: Correlation analysis of RPR in all environments; Figure S5: Comparative analysis of QTL results for SD and RPR. Table S1: Primer sequences used in this study; Table S2: QTL clusters for SD and RPR in four environments; Table S3: Maize cellwall biosynthesis genes in the confidence intervals of RPR-related QTLs.
