# Supplementary material for: Genetic Architecture of Maize Stalk Diameter and Rind Penetrometer Resistance in a Recombinant Inbred Line Population
Source: Genes (Basel). 2022 Mar 24;13(4):579. doi: 10.3390/genes13040579 (PMC9032882; doi:10.3390/genes13040579)
Supplement: Supplementary file 1 [file genes-13-00579-s001.zip › Figures S1-S5.pdf]

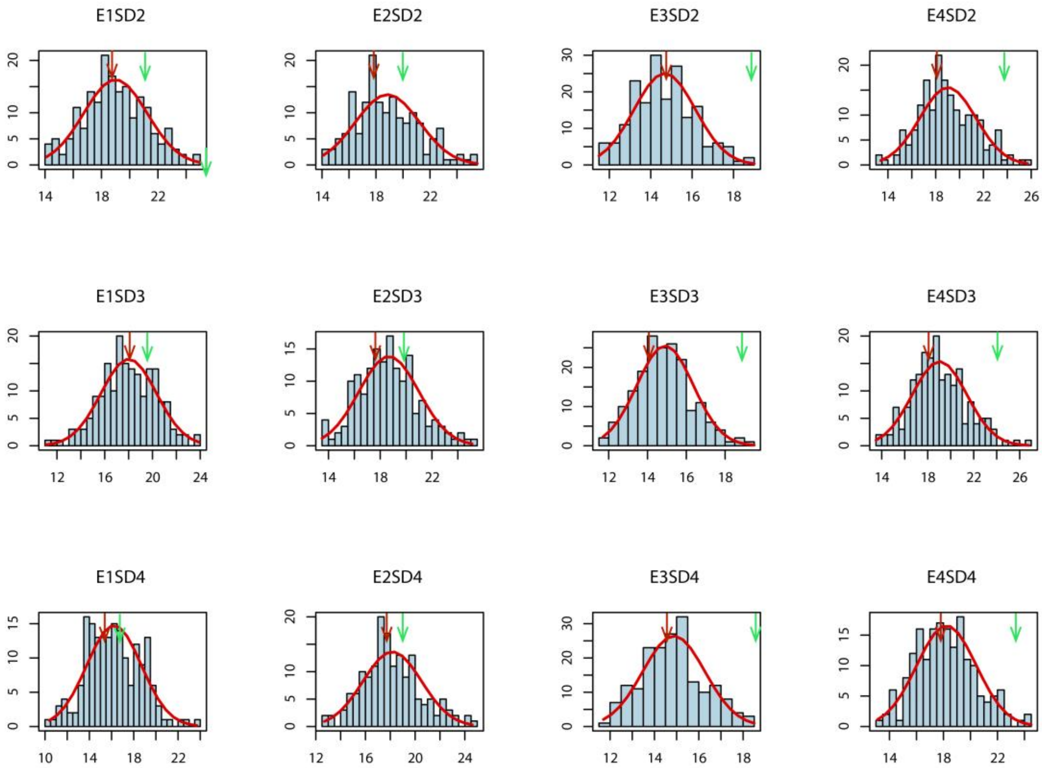

Figure S1. Frequency distributions of SD in the RIL population in four environments. Red and green arrows represent the mean value of the parents YS501 and LDC-1, respectively.

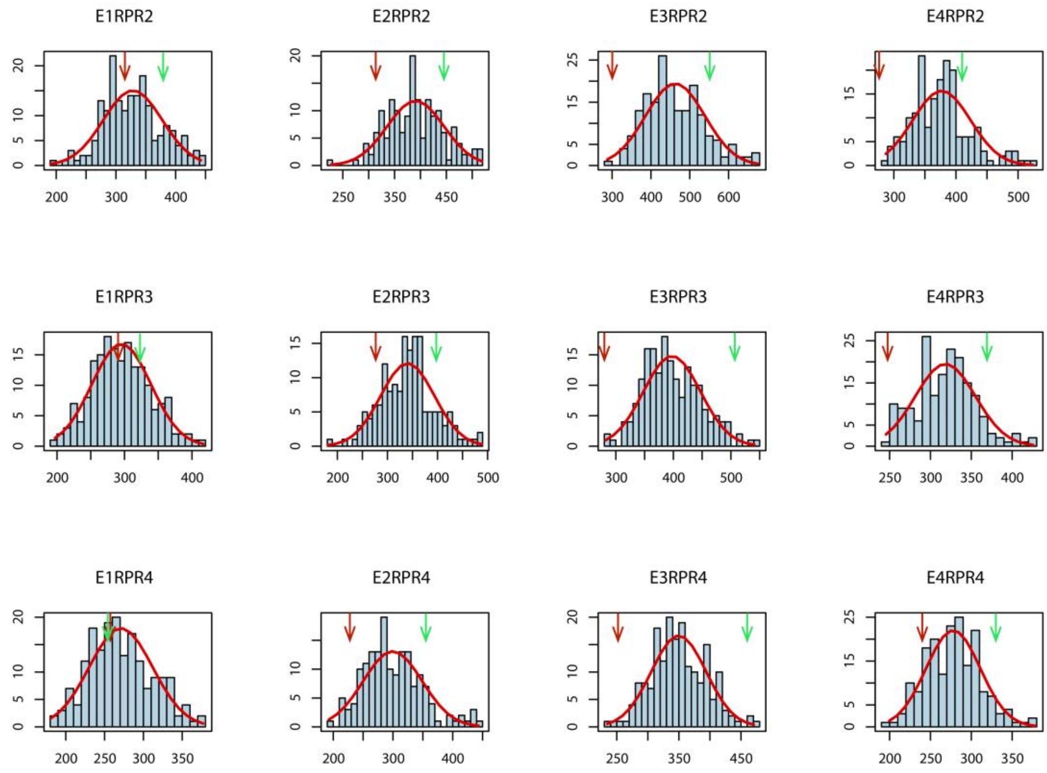

Figure S2. Frequency distributions of RPR in the RIL population in four environments. Red and green arrows represent the mean value of the parents YS501 and LDC-1, respectively.

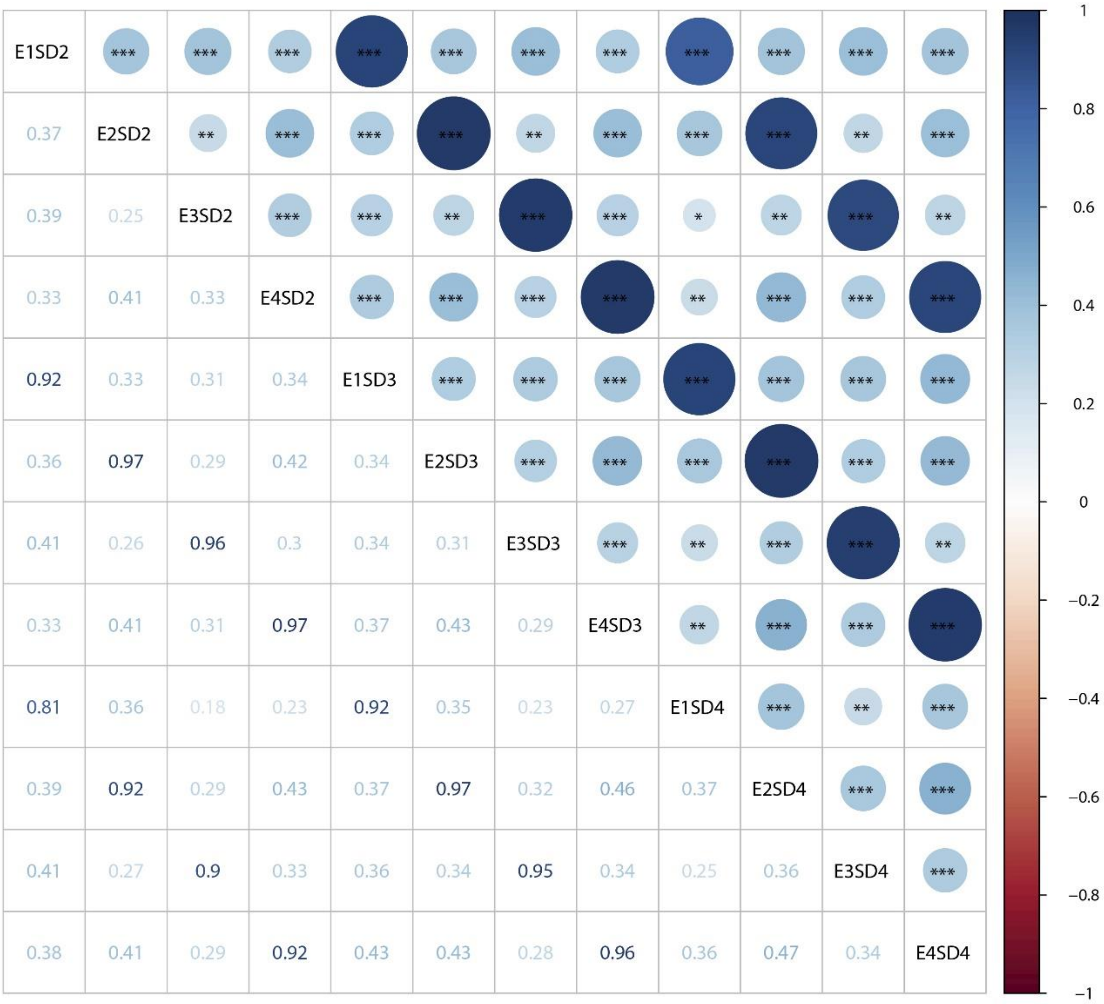

Figure S3. Correlation analysis of SD in all environments. Size and color of the dots in the upper triangle indicate the degree and direction of correlation, respectively; values in the lower triangle indicate Pearson's correlation coefficient. *, **, ***: the correlation is significant at the 0.05, 0.01, and 0.001 level, respectively.

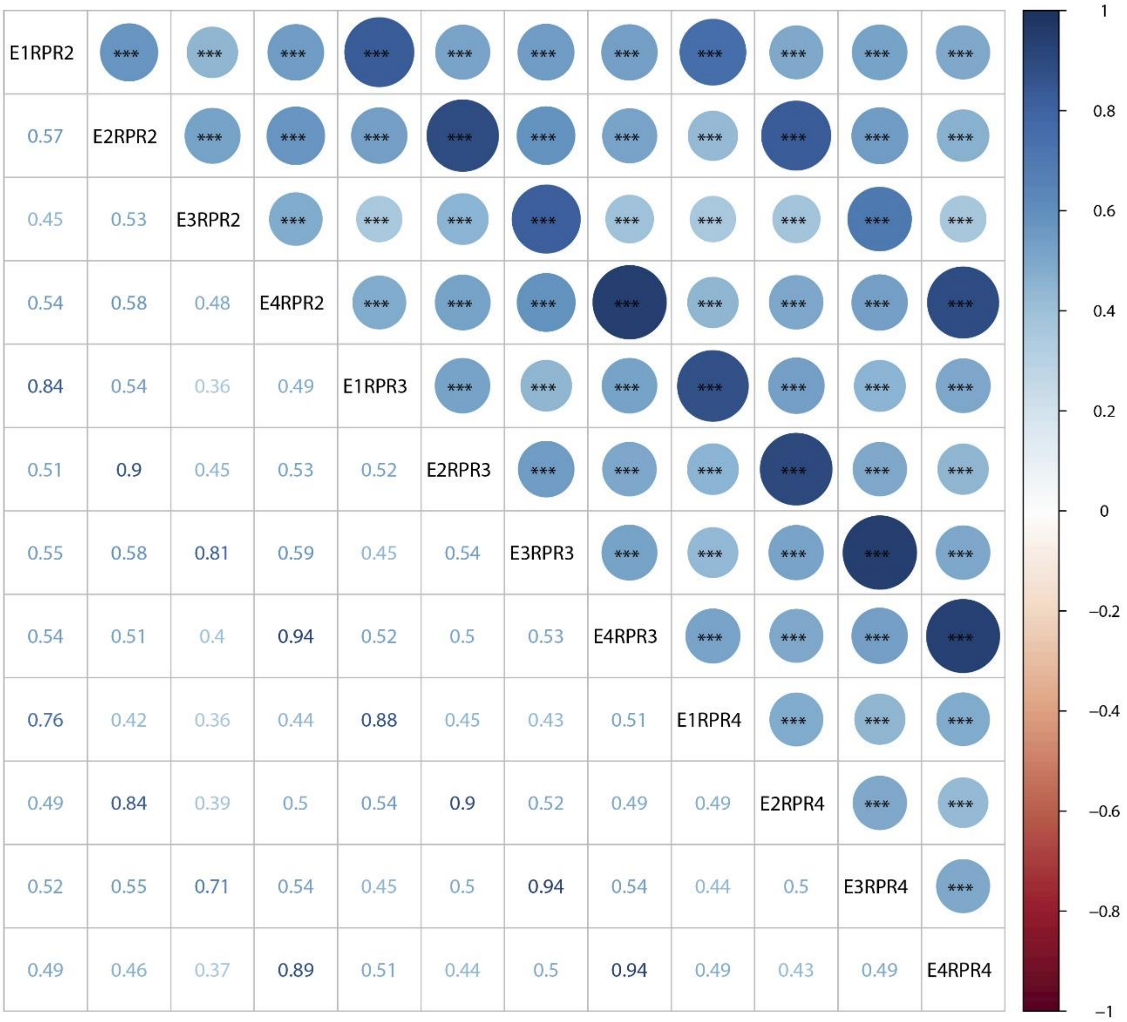

Figure S4. Correlation analysis of RPR in all environments. Size and color of the dots in the upper triangle indicate the degree and direction of correlation, respectively; the values in the lower triangle indicate Pearson's correlation coefficient. *, **, ***: the correlation is significant at the 0.05, 0.01, and 0.001 level, respectively.

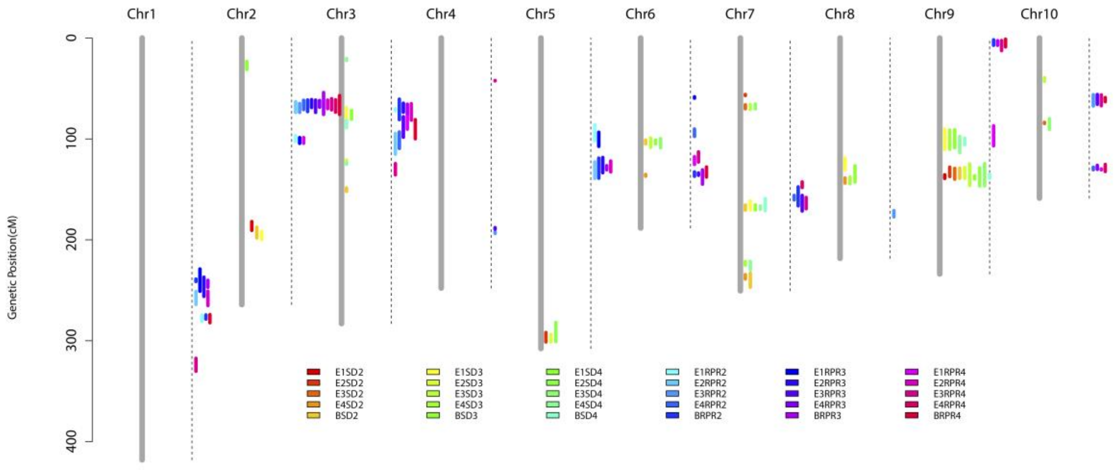

Figure S5. Comparative analysis of QTL results for SD and RPR. Colored bars represent QTLs associated with different traits; E1, E2, E3, E4, and B represent the corresponding environment or BLUP model.The dashed line next to each chromosome separates the QTL mapping results of SD and RPR. The length of the colored bar indicates the 1.5-LOD QTL support interval.
